# *N‐*acetylcysteine attenuates the development of cardiac fibrosis and remodeling in a mouse model of heart failure

**DOI:** 10.14814/phy2.12757

**Published:** 2016-04-13

**Authors:** Beverly Giam, Po‐Yin Chu, Sanjaya Kuruppu, A. Ian Smith, Duncan Horlock, Helen Kiriazis, Xiao‐Jun Du, David M. Kaye, Niwanthi W. Rajapakse

**Affiliations:** ^1^ Baker IDI Heart and Diabetes InstituteMelbourneAustralia; ^2^ Central Clinical SchoolMonash UniversityMelbourneAustralia; ^3^ Department of BiochemistryMonash UniversityMelbourneAustralia; ^4^ Department of MedicineMonash UniversityMelbourneAustralia; ^5^ Department of PhysiologyMonash UniversityMelbourneAustralia

**Keywords:** Fibrosis, heart failure, *N‐*acetylcysteine, oxidative stress

## Abstract

Oxidative stress plays a central role in the pathogenesis of heart failure. We aimed to determine whether the antioxidant *N‐*acetylcysteine can attenuate cardiac fibrosis and remodeling in a mouse model of heart failure. Minipumps were implanted subcutaneously in wild‐type mice (*n *= 20) and mice with cardiomyopathy secondary to cardiac specific overexpression of mammalian sterile 20‐like kinase 1 (MST‐1; *n *= 18) to administer *N‐*acetylcysteine (40 mg/kg per day) or saline for a period of 8 weeks. At the end of this period, cardiac remodeling and function was assessed via echocardiography. Fibrosis, oxidative stress, and expression of collagen types I and III were quantified in heart tissues. Cardiac perivascular and interstitial fibrosis were greater by 114% and 209%, respectively, in MST‐1 compared to wild type (*P* ≤ 0.001). In MST‐1 mice administered *N‐*acetylcysteine, perivascular and interstitial fibrosis were 40% and 57% less, respectively, compared to those treated with saline (*P* ≤ 0. 03). Cardiac oxidative stress was 119% greater in MST‐1 than in wild type (*P* < 0.001) and *N‐*acetylcysteine attenuated oxidative stress in MST‐1 by 42% (*P* = 0.005). These data indicate that *N‐*acetylcysteine can blunt cardiac fibrosis and related remodeling in the setting of heart failure potentially by reducing oxidative stress. This study provides the basis to investigate the role of *N‐*acetylcysteine in chronic heart failure.

## Introduction

Heart failure (HF) is increasing at a rapid rate worldwide in part due to an ageing population and high prevalence of obesity, diabetes, and hypertension. While the pathogenesis of HF is complex and multifactorial, there is direct experimental and clinical data to indicate that oxidative stress plays a central role in its pathogenesis (Seddon et al. 2007). Oxidative stress can contribute to the development of cardiac remodeling and fibrosis (Tsutsui et al. 2011; Tham et al. 2015) and these cardiac adaptations can ultimately lead to failing of the myocardium (Tsutsui et al. 2011). For example, oxidative stress has been demonstrated to increase the expression of cardiac collagen types I and IV and fibronectin and impair cardiac contractility in diabetic rats (Aragno et al. 2008). There is also evidence that oxidative stress plays a role in the development of angiotensin II‐dependent cardiac fibrosis in rats (Zhao et al. 2008; Worou et al. 2011) and mice (Li et al. 2013). In this context, it is of interest to note that the presence of oxidative stress is widely documented in patients with chronic HF regardless of the underlying etiology (McMurray et al. 1993; Thomson et al. 2009). Consequently, therapeutic strategies which target oxidative stress have received much attention in the setting of HF (Thomson et al. 2009).

There is evidence that left ventricular glutathione content is reduced in HF (Adamy et al. 2007), and of particular interest, restoring the glutathione content via oral supplementation of its precursor, *N‐*acetylcysteine (NAC), reduced oxidative stress, and restored HF‐related cardiac damage and function in rats (Adamy et al. 2007). In addition to reducing oxidative stress, NAC treatment reduced the expression of the pro‐inflammatory cytokine tumor necrosis factor alpha (TNF‐*α*) and its receptor expression in these rats (Adamy et al. 2007). Consistent with this, NAC treatment reduced serum levels of TNF‐*α*, transforming growth factor *β* (TGF‐*β*), matrix metalloproteinase 9 (MMP‐9), and MMP‐2 in patients with acute myocardial infarction (Talasaz et al. 2013). These cytokines and enzymes play an important role in the development of cardiac fibrosis, remodeling, and subsequent cardiac dysfunction (Peng et al. 2002; Bourraindeloup et al. 2004; Skyschally et al. 2007). Therefore, NAC is likely to simultaneously target multiple mechanisms which underpin the progressive worsening of cardiac function in HF. In particular, cardiac fibrosis remains an independent risk factor for cardiomyopathy‐related morbidity and mortality (Leyva et al. 2012; Gulati et al. 2013) and treatment interventions that effectively target cardiac fibrosis remain an unmet clinical need. Consequently, the aim of the current study was to determine whether NAC can exert cardioprotective effects in the setting of HF. We hypothesized that NAC can reduce cardiac oxidative stress and thereby attenuate cardiac remodeling and fibrosis in HF.

## Methods

### Ethical approval

All study protocols were approved by the Alfred Medical Research and Education Precinct Animal Ethics Committee. All experiments were conducted in accordance with the Australian Code of Practice for Care and Use of Animals for Scientific Purposes.

### Animals

Male mice (18 transgenic and 20 C57BL/6 mice) were allowed food and water ad libitum. Transgenic mammalian sterile 20‐like kinase 1 (*Mst1*) mice with cardiac‐specific overexpression MST1 under the control of *α*‐myosin heavy chain promoter were bred as previously described (Yamamoto et al. 2003). The cardiac specific overexpression of Mst1, a key mediator of apoptosis, drives the development of heart failure in MST‐1 transgenic mice (Yamamoto et al. 2003; Chu et al. 2010). Compared to WT controls, left ventricular ejection fraction and fractional shortening were less by 32% and 42%, respectively, in 76 days (approximately 10 weeks) old MST‐1 mice (Yamamoto et al. 2003). In addition, lung weight and liver weight were greater by 13% and 16%, respectively, in 76 days old MST‐1 compared to age‐matched WT (Yamamoto et al. 2003) indicating MST‐1 mice have evidence of organ congestion, which is a salient feature of heart failure. We found that these mice develop extensive cardiac fibrosis by 13 weeks of age (Chu et al. 2010). Collectively, these data provide evidence that MST‐1 mice develop many features commonly present in human heart failure by approximately 10 weeks of age (Gulati et al. 2013)**.** Therefore, in the present study, we assessed the effects of NAC on cardiac fibrosis, remodeling, and function in 14‐week‐old MST‐1 mice.

### Minipump implantation

At 6 weeks of age, WT and MST‐1 mice were randomly allocated to receive either NAC (40 mg/kg per day; Sigma‐Aldrich, Sydney, Australia) or saline vehicle via subcutaneously implanted minipumps (Alzet Model 2004; Alzet Corporation, Curpertino, CA). Minipumps were implanted in mice under isoflurane anesthesia (3–4.5% induction and 1.5–2% maintenance delivered through a mask) as previously described by us (Konstantinidis et al. 2014). Administration of NAC or saline continued for a period of 8 weeks and terminal experiments were performed during the 8th week when mice were 14 weeks of age. All minipumps were weighed before implantation and reweighed after explantation at the end of the treatment protocol to ensure that the minipump was functional and there was successful delivery of the respective treatment.

### Echocardiography

Isoflurane (3–4.5% induction and 1.5–2% maintenance delivered through a mask) was used to anesthetize mice and echocardiography was performed as we have previously described (Chu et al. 2011; Rajapakse et al. 2015). In brief, m‐mode two‐dimensional echocardiography was performed using a Philips iE33 echocardiography system (Royal Philips Electronics, Amsterdam, Netherlands) with a 15‐MHz linear array transducer. Ultrasound images of the heart were obtained by gently applying the probe to the chest. Image analysis was performed in a blinded fashion, and derived echocardiography parameters include diastolic interventricular septum (IVSd), systolic interventricular septum (IVSs), left ventricle diastolic posterior wall thickness (LVPWd), left ventricle systolic posterior wall thickness (LVPWs), LV end‐diastolic dimension (LVDD), LV end‐systolic dimension (LVSD), and heart rate. The following functional measurements were then calculated: LV end‐diastolic volume (LVDV) = [(4/3) × 3.14 × LVDD^2^], LV end‐systolic volume (LVSV) = [(4/3) × 3.14 × LVSD^2^], stroke volume (SV) = LVDV‐LVSV, cardiac output = HR × SV, ejection fraction = [(LVDV‐LVSV)/LVDV × 100%], and fractional shortening (FS) ([(LVDD‐LVSD)/LVDD] × 100%) using the above‐mentioned echocardiographic parameters. LV mass was also calculated as (LVDD + LVPWd + IVSd)^3^‐ [LVDD]^3^ and used as a measure of cardiac hypertrophy.

### Blood pressure measurement via cardiac catheterization

Two days after performing echocardiography, mice were reanesthetized as described above and arterial and left ventricular pressure were measured via cardiac catheterization as we have previously described (Du et al. 2000; Chu et al. 2011). Briefly, 1.4F catheter (Millar Instruments, Houston, TX) was inserted into the left ventricle via the right carotid artery. Recordings of blood pressure were taken for at least 5 min or until we obtained a stable blood pressure trace.

### Collection of tissues

After aortic and left ventricular blood pressure measurements were completed, mice were killed by rapidly removing the heart while they were under deep isoflurane anesthesia. Hearts were cut into three sections in the horizontal short axis plane. The apex of the heart was snap frozen in liquid nitrogen for later analysis of collagen types I and III, brain natriuretic peptide (BNP) and *β*‐myosin heavy chain (*β*‐MHC) mRNA expression. The mid section of the heart was stored in formalin and then embedded in paraffin for later analysis of fibrosis. Base of the heart was embedded in OCT and then frozen in dry ice for quantification of oxidative stress via dihydroethidium (DHE) staining.

### Assessment of cardiac fibrosis

Cardiac fibrosis was assessed using Masson Trichrome staining as previously described by us (Chu et al. 2011). 4‐*μ*m thick sections of cardiac tissue were sectioned and for each section and measurement of fibrosis, 10 fields were chosen at random and imaged under light microscopy using the Olympus BH2 microscope at ×40 magnification. Fibrosis was then quantified using ImagePro Plus software (Adept Electronic Solutions Pty Ltd, Moorabin, Australia).

### Quantification of collagen type I, collagen type III, cardiac brain natriuretic peptide and *β*‐Myosin heavy chain expression by PCR

Cardiac mRNA expression of collagen types I and III, BNP and *β*‐MHC was assessed by PCR as we have previously described (Rajapakse et al. 2015). The following primers were used for (5′–3′) 18S (Forward: TTC GAG GCC CTG TAA TTG GA and Reverse: GCA GCA ACT TTA ATA TAC GCT ATT GG), CAT1 (forward: 5′‐CAT GCC CCG AGT TAT CTA TGC‐3′ and (reverse: TTT TGG TCC TAT TGT TGA TTT TGG‐3′) collagen I (Forward: GGA GAT GAT GGG GAA GCT G and Reverse: AAT CCA CGA GCA CCC TGA), collagen III (Forward: GGA ATG GAG CAA GAC AGT CTT TG and Reverse: TGC GAT ATC TAT GAT GGG TAG TCT CA), *β*‐Myosin heavy chain (*β*‐MHC, Forward: TCT CTT GCT GTT TCC TTA CTT GCT A and reverse: GTA CTC CTC TGC TGA GGC TTC CT) and cardiac BNP (Forward: CCT GGC CCA TCG CTT CT and Reverse: CAT CTG GGA CAG CAC CTT CA).

### Assessment of cardiac oxidative stress

DHE (Sigma Aldrich, Sydney, Australia) staining was used to detect superoxide production within the heart. Frozen heart tissue (10 *μ*m thick) was sectioned and incubated with DHE (10 *μ*mol/L) at 37°C for 45 min in a light‐protected and humidified chamber. Nuclei were then co‐stained with Hoerscht (1 *μ*mol) for 10 min in a light‐protected chamber. Ten fluorescent images per section of Hoerscht and DHE staining were obtained using Nikon A1R confocal microscope at a magnification of ×20. Fiji software was used to create a mask around each nucleus based on images with Hoerscht fluorescence. This mask was then applied to the corresponding fluorescent image of DHE, and mean pixel intensity within the masked areas was quantified.

### Statistics

Data are expressed as mean ± SEM. Graphpad Prism Version 6 (San Diego, CA) was used for all analyses. One‐way ANOVA followed by Tukey post hoc tests were used for multiple comparisons. Two‐tailed *P* ≤ 0.05 was considered to be statistically significant.

## Results

### Cardiac hypertrophy

**Figure 1:**
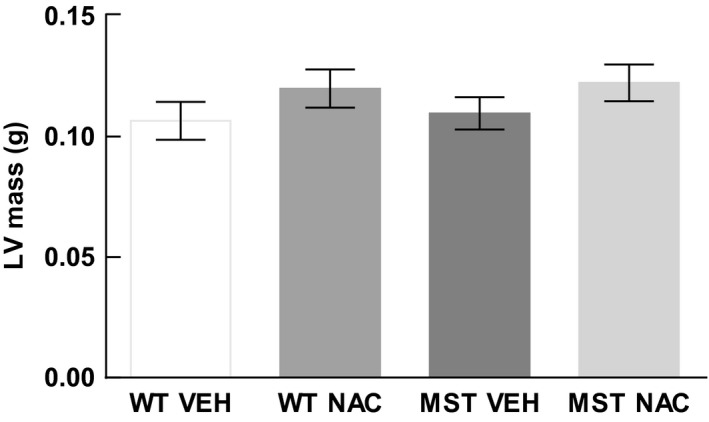
LV mass of WT and MST‐1 mice treated with NAC or saline vehicle. LV mass of WT and MST‐1 mice receiving saline were not significantly different. WT,wild‐type mice; MST‐1, transgenic mice with cardiac‐specific overexpression of MST‐1; NAC,* N‐*acetylcysteine; VEH, vehicle.

LV mass did not significantly differ between WT and MST‐1 mice treated with saline vehicle (*P* *= *0.99; Fig. 1).

### Echocardiography

**Figure 2:**
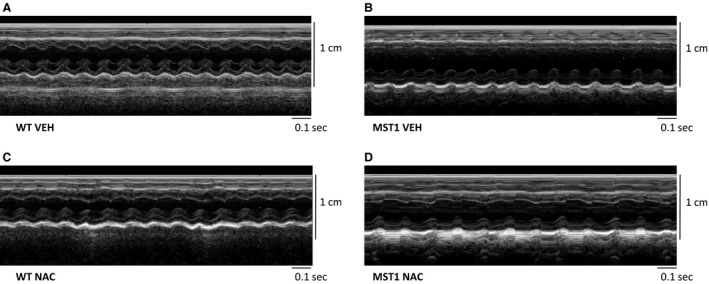
A–D Representative photomicrographs of raw echocardiographic traces of WT and MST‐1 mice treated with NAC or saline vehicle. MST‐1 mice receiving saline had thinner interventricular septum walls, thinner left ventricular posterior walls, and bigger left ventricular dimensions compared to WT receiving saline. Treatment with NAC in MST‐1 mice attenuated the heart failure ‐dependent changes in the thickness of the interventricular septum. WT, wild‐type mice; MST‐1, transgenic mice with cardiac‐specific overexpression of MST‐1; NAC,* N‐*acetylcysteine; VEH, vehicle.

The thickness of the interventricular septum was 29% and 23% less during systole and diastole, respectively, in saline‐treated MST‐1 mice compared to saline‐treated WT mice (*P* ≤ 0.02; Table 1; Fig. 2A and B). The thickness of the interventricular septum during systole and diastole was greater by 28% and 41% in MST‐1 mice receiving NAC, compared to those receiving saline (*P* ≤ 0.01; Table 1; Fig. 2B and D). Left ventricular end‐systolic dimension was 26% greater in MST‐1 mice administered saline compared to WT mice administered saline (*P* = 0.02; Table 2; Fig. 2A and B) and NAC had minimal effect on this parameter in both genotypes (*P* ≥ 0.63; Fig. 2). Ejection fraction and fractional shortening were 29% and 38% less, respectively, in MST‐1 mice receiving saline compared to WT mice receiving saline (*P *< 0.01; Table 2), and NAC treatment had minimal effect on ejection fraction and fractional shortening in MST‐1 mice (*P* > 0.76; Table 2). Other echocardiographic indices of left ventricular function and size were not significantly different between the two genotypes of mice, and NAC treatment had no detectable effects on these measurements (Tables 1, 2).

**Table 1:** Echocardiographic features of cardiac remodeling in WT and MST‐1 mice at 14 weeks of age

	WT Vehicle	WT NAC	MST‐1 Vehicle	MST‐1 NAC
Group size	10	10	8	10
IVSd, mm	0.75 ± 0.03	0.81 ± 0.04	0.58 ± 0.03*	0.81 ± 0.05 †††
IVSs, mm	1.18 ± 0.05	1.32 ± 0.04	0.84 ± 0.04***	1.08 ± 0.05†
LVPWd, mm	0.80 ± 0.03	0.87 ± 0.04	0.85 ± 0.04	0.87 ± 0.05
LVPWs, mm	1.08 ± 0.06	1.14 ± 0.05	1.01 ± 0.03	1.06 ± 0.07
Mean wall thickness, mm	0.77 ± 0.02	0.83 ± 0.03	0.72 ± 0.02	0.81 ± 0.05†

Values are means ± SEM.

IVS, interventricular septum (anterior wall); LVPW, LV posterior wall; Mean wall thickness, (IVSd + LVPWd)/ 2; WT, Wild type; MST‐1, transgenic mice overexpressing MST‐1 in a cardiac‐specific manner; NAC, *N‐*acetylcysteine.

*P < 0.05, ***P < 0.001 versus WT vehicle.

^†^ *P *< 0.05,^†††^ *P *< 0.001 versus MST‐1 vehicle.

**Table 2:** Echocardiographic features of cardiac function in WT and MST‐1 mice at 14 weeks of age

	WT Vehicle	WT NAC	MST‐1 Vehicle	MST‐1 NAC
Group size	10	10	8	10
HR, bpm	542.7 ± 19.45	541.2 ± 20.17	576.9 ± 27.53	522.4 ± 19.40
LVDD, mm	3.97 ± 0.15	4.00 ± 0.09	4.31 ± 0.13	4.08 ± 0.19
LVSD, mm	2.79 ± 0.16	2.66 ± 0.14	3.51 ± 0.13*	3.23 ± 0.19
LVDV, *μ*L	33.93 ± 3.80	33.87 ± 2.34	42.67 ± 4.17	37.42 ± 4.53
LVSV, *μ*L	12.32 ± 2.03	10.64 ± 1.43	23.37 ± 2.79*	19.27 ± 3.12
SV, *μ*L	21.62 ± 2.10	23.23 ± 1.14	19.30 ± 1.70	18.15 ± 1.91
CO, mL/min	11.69 ± 1.20	12.49 ± 0.55	11.06 ± 0.95	9.56 ± 1.18
EF, %	65 ± 3	70 ± 3	46 ± 2***	51 ± 3
LVFS, %	30 ± 2	34 ± 2	19 ± 1***	21 ± 2

Values are means ± SEM.

HR, heart rate; LVDD, LV diastolic dimension; LVSD, LV systolic dimension; LVDV, LV end‐diastolic volume; LVSV, LV end‐systolic volume; SV, stroke volume; CO, cardiac output; EF, ejection fraction; LVFS, LV fractional shortening; WT, Wild type; MST‐1, transgenic mice overexpressing MST‐1 in a cardiac‐specific manner; NAC, *N‐*acetylcysteine.

*P < 0.05, ***P < 0.001 versus WT vehicle.

### Blood pressure

**Figure 3:**
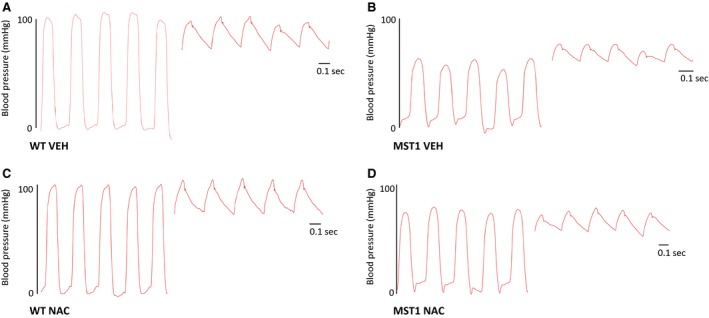
A–D Raw data traces of blood pressure in WT and MST‐1 mice treated with NAC or saline vehicle. The segments on the left are traces of left ventricular blood pressure. Segments on the right are traces of arterial pressure. MST‐1 mice receiving saline had lower blood pressure compared to WT receiving saline. Treatment with NAC in MST‐1 mice had no effect on blood pressure. WT,wild‐type mice; MST‐1, transgenic mice with cardiac‐specific overexpression of MST‐1; NAC,* N‐*acetylcysteine; VEH, vehicle.

**Figure 4:**
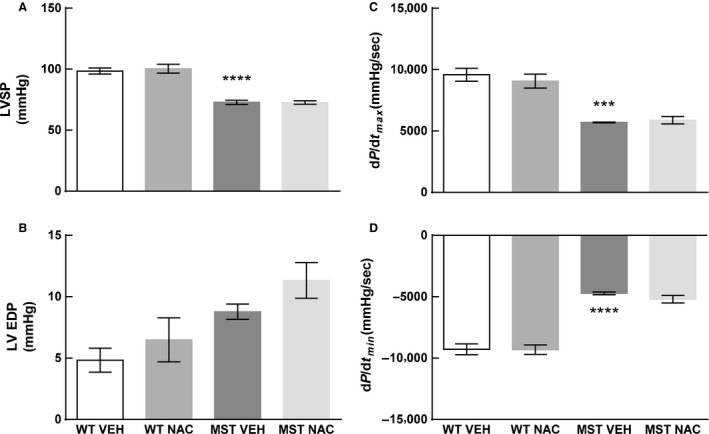
(A) Left ventricular systolic pressure (B) left ventricular end diastolic pressure (C) maximum rate of increase in LV pressure (D) minimum rate of decay in LV pressure in WT and MST‐1 mice treated with NAC or saline vehicle (*n *= 4–5). Left ventricular systolic pressure and the maximum rate of increase in left ventricular pressure were less in MST‐1 mice compared to WT mice receiving saline. Left ventricular end‐diastolic pressure was not significantly different between MST‐1 mice receiving saline and WT receiving saline. Minimum rate of decay in left ventricular pressure was greater in MST‐1 mice receiving saline compared to WT controls. Treatment with NAC had no effect on left ventricular blood pressure. Data are mean ± SEM. ****P < 0.0001 versus WT vehicle, ***P = 0.0001. LVSP, left ventricular systolic pressure; LVEDP, left ventricular end diastolic pressure; d*P*/d*t*_max_, maximal rate of increase in left ventricular pressure; d*P*/d*t*_min_, minimal rate of decay in left ventricular pressure; WT, wild‐type mice; MST‐1, transgenic mice with cardiac‐specific overexpression of MST‐1; NAC,* N‐*acetylcysteine; VEH, vehicle.

**Figure 5:**
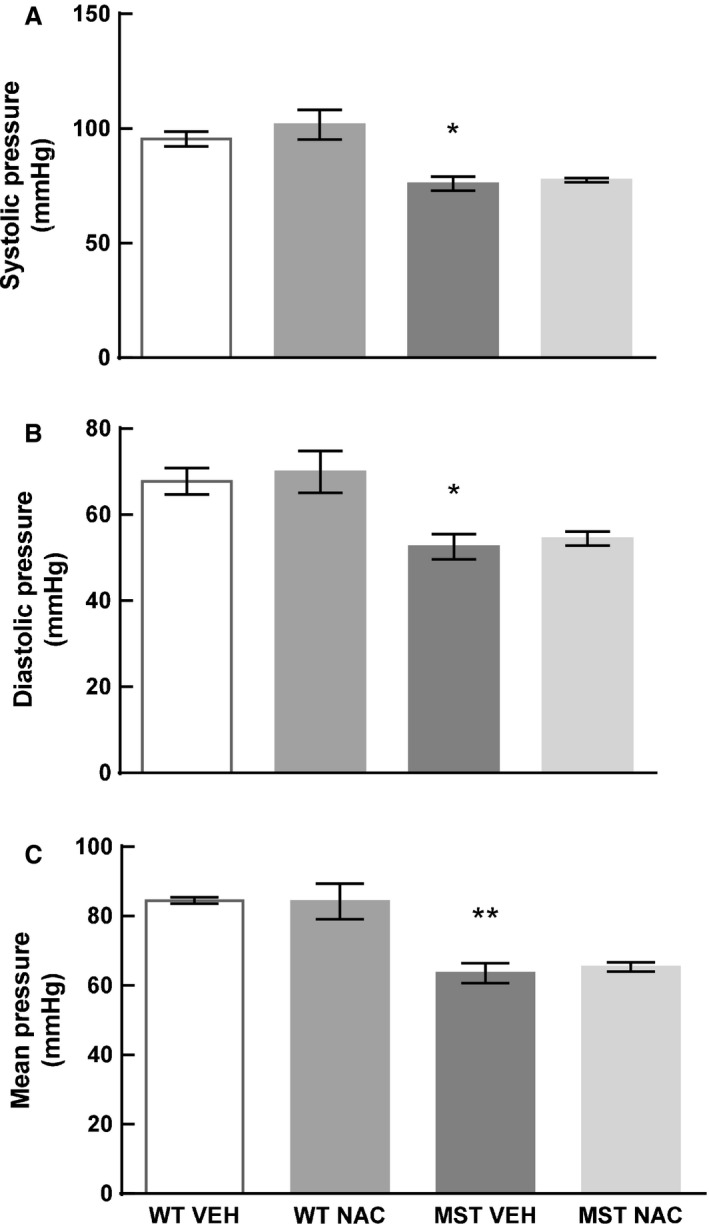
(A) Systolic pressure (B) diastolic pressure and (C) mean arterial pressure in WT and MST‐1 mice treated with saline vehicle or NAC (*n*=4). Arterial blood pressure was less in MST‐1 mice receiving saline compared to WT controls. Treatment with NAC had no effect on arterial blood pressure. Data are mean ± SEM. *P < 0.05, **P < 0.01 versus WT vehicle. WT, wild‐type mice; MST‐1, transgenic mice with cardiac‐specific overexpression of MST‐1; NAC,* N‐*acetylcysteine; VEH, vehicle.

Left ventricular systolic pressure as well as maximum rate of increase (d*P*/d*t*_max_) and decay of left ventricular pressure (d*P*/d*t*_min_) were less in MST‐1 mice administered saline compared to WT mice administered saline (*P* < 0.001; Figs. 3, 4A, C and D). In both genotypes of mice, these measurements were not significantly affected by NAC treatment (*P* *≥ *0.79; Figs. 3, 4A, C and D). Left ventricular end‐diastolic pressure was not significantly different between WT and MST‐1 mice treated with saline vehicle (*P = *0.23; Fig. 4B). Mean arterial pressure, systolic arterial pressure, and diastolic arterial pressure were less in MST‐1 mice compared to WT mice (*P *≤ 0.007; Figs. 3, 5). In both genotypes, NAC had minimal effect on blood pressure (*P ≥ *0.52; Figs. 3, 5).

### Cardiac fibrosis

**Figure 6:**
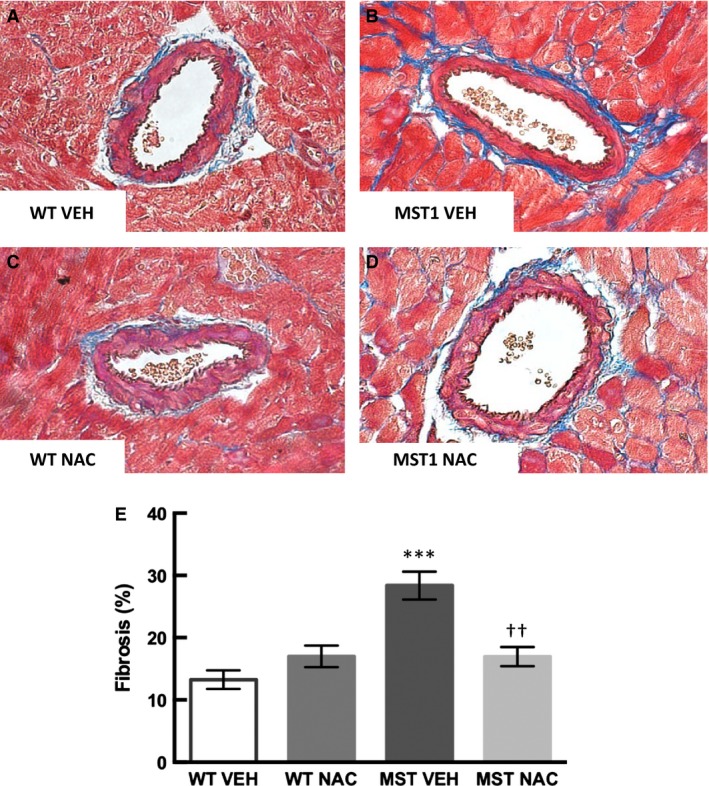
(A–D) Representative photomicrographs of Masson trichrome‐stained cardiac sections of WT and MST‐1 mice treated with NAC or saline vehicle. Blue staining indicates perivasuclar fibrosis in the heart (E) Percentage of cardiac perivascular fibrosis in NAC or saline vehicle treated WT and MST‐1 mice (*n *= 5–6). Cardiac perivascular fibrosis in MST‐1 mice was significantly greater than in WT controls. Treatment with NAC prevented the development of perivascular fibrosis in MST‐1 mice. Data are mean ± SEM. ****P < 0.0001 versus WT vehicle. ††*P* = 0.001 versus MST‐1 vehicle. WT, wild‐type mice; MST‐1, transgenic mice with cardiac‐specific overexpression of MST‐1; NAC,* N‐*acetylcysteine; VEH, vehicle.

**Figure 7:**
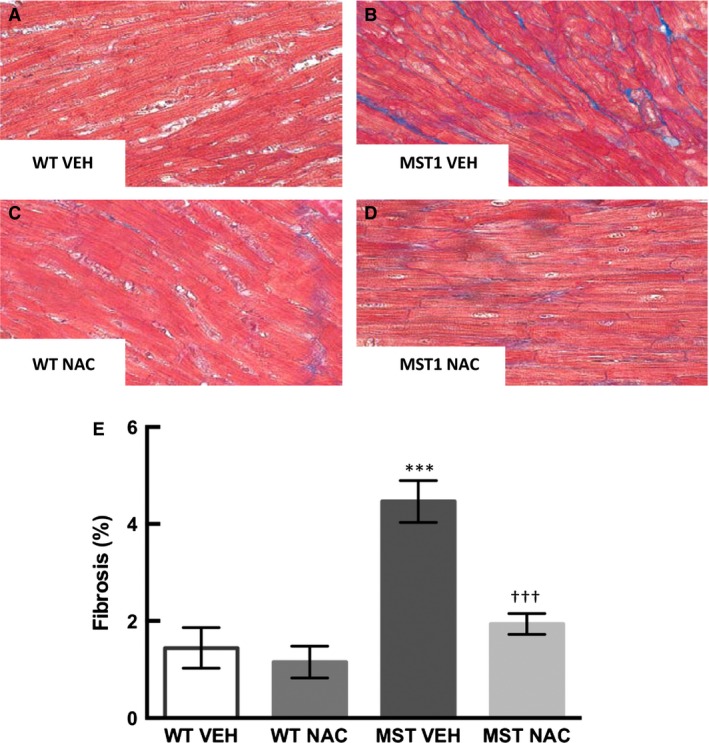
(A–D) Representative photomicrographs of Masson trichrome‐stained cardiac sections of WT and MST‐1 mice treated with NAC or saline vehicle. Blue staining indicates interstitial fibrosis in the heart (E): Percentage of cardiac interstitial fibrosis in NAC or saline vehicle treated WT and MST‐1 mice (*n *= 5–6). Cardiac interstitial fibrosis in MST‐1 mice was significantly greater than in WT controls. Treatment with NAC prevented the development of interstital fibrosis in MST‐1 mice. Data are mean ± SEM. ****P < 0.0001 versus WT vehicle. †††*P* < 0.001 versus MST‐1 vehicle. WT,wild‐type mice; MST‐1, transgenic mice with cardiac‐specific overexpression of MST‐1; NAC,* N‐*acetylcysteine; VEH, vehicle.

Perivascular and interstitial fibrosis was 114% and 209% greater, respectively, in saline‐treated MST‐1 mice compared to saline‐treated WT mice (*P* < 0.001; Figs. 6, 7). In NAC‐treated MST‐1 mice, there was 40% less perivascular fibrosis and 57% less interstitial fibrosis than saline vehicle‐treated MST‐1 mice (*P *≤ 0.001; Figs. 6, 7). Following 8 weeks of NAC treatment, perivascular and interstitial fibrosis in MST‐1 mice was comparable to levels of fibrosis observed in saline‐treated WT mice (*P* ≥ 0.46; Figs. 6, 7).

### Cardiac collagen expression

**Figure 8:**
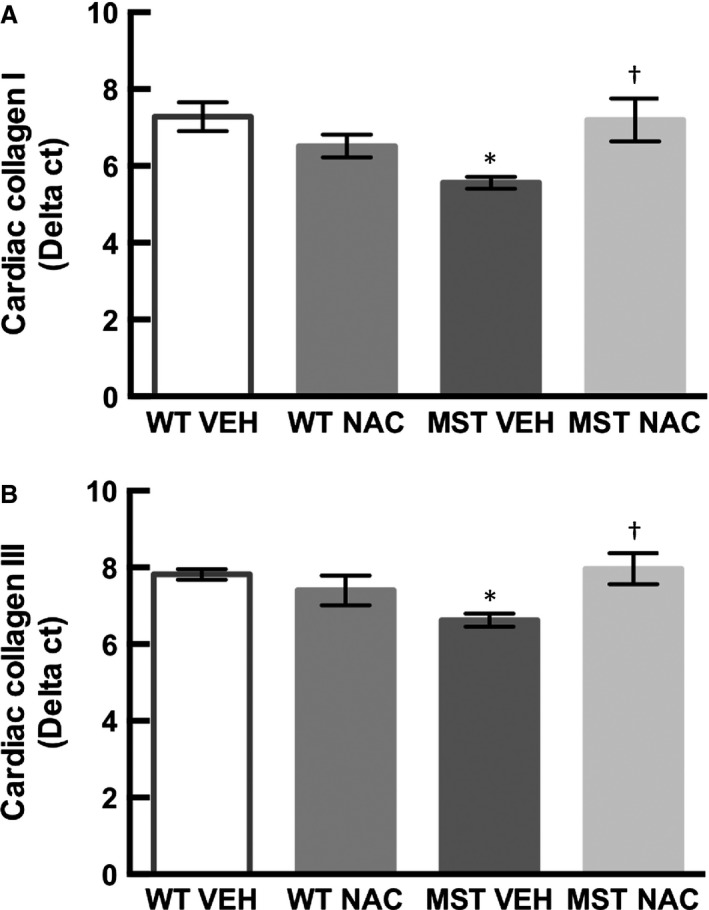
Cardiac mRNA expression of (A) collagen type I and (B) collagen type III in WT and MST‐1 mice treated with NAC or saline vehicle (*n *= 6–7). Cardiac mRNA expression of collagen type I and III were less in MST‐1 mice receiving saline compared to WT controls. Treatment with NAC restored cardiac mRNA expression of collagen type I and III. Data are mean ± SEM. *P < 0.05 versus WT vehicle. †*P* < 0.05 versus MST‐1 vehicle. WT,wild‐type mice; MST‐1, transgenic mice with cardiac‐specific overexpression of MST‐1; NAC,* N‐*acetylcysteine; VEH, vehicle.

Expression of cardiac collagen type I and type III mRNA was greater in MST‐1 mice administered saline compared to WT mice administered saline (*P* ≤ 0.03; Fig. 8). Expression of cardiac collagen type I and type III was significantly less in MST‐1 mice receiving NAC compared to those receiving saline (*P* ≤ 0.03; Fig. 8).

### Cardiac BNP and *β*‐MHC expression

**Figure 9:**
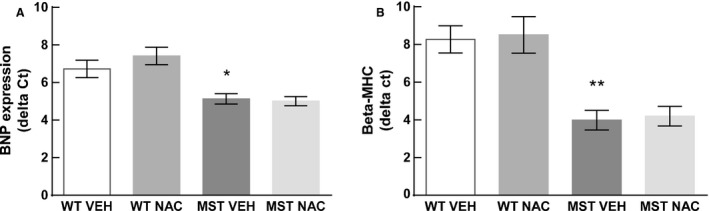
Cardiac mRNA expression of (A) BNP and (B) *β*‐MHC in WT and MST‐1 mice treated with NAC or saline vehicle (*n *=* *6–7). Cardiac mRNA expression of BNP and *β*‐MHC were less in MST‐1 mice receiving saline compared to WT receiving saline. Treatment with NAC had minimal effect on the cardiac mRNA expression of BNP and *β*‐MHC. Data are mean ± SEM. **P < 0.01, *P < 0.05 versus WT vehicle. BNP, brain natriuretic peptide, *β*‐MHC,* β* myosin heavy chain. WT,wild‐type mice; MST‐1, transgenic mice with cardiac‐specific overexpression of MST‐1; NAC,* N‐*acetylcysteine; VEH, vehicle.

Consistent with augmented cardiac fibrosis and collagen expression in MST‐1 mice, expression of cardiac BNP and *β*‐MHC was also greater in this genotype compared to WT mice (*P* ≤ 0.03; Fig. 9). In both genotypes, NAC treatment had minimal effect on cardiac BNP and *β*‐MHC expression (*P ≥ *0.56; Fig. 9).

### Assessment of cardiac oxidative stress via DHE

**Figure 10:**
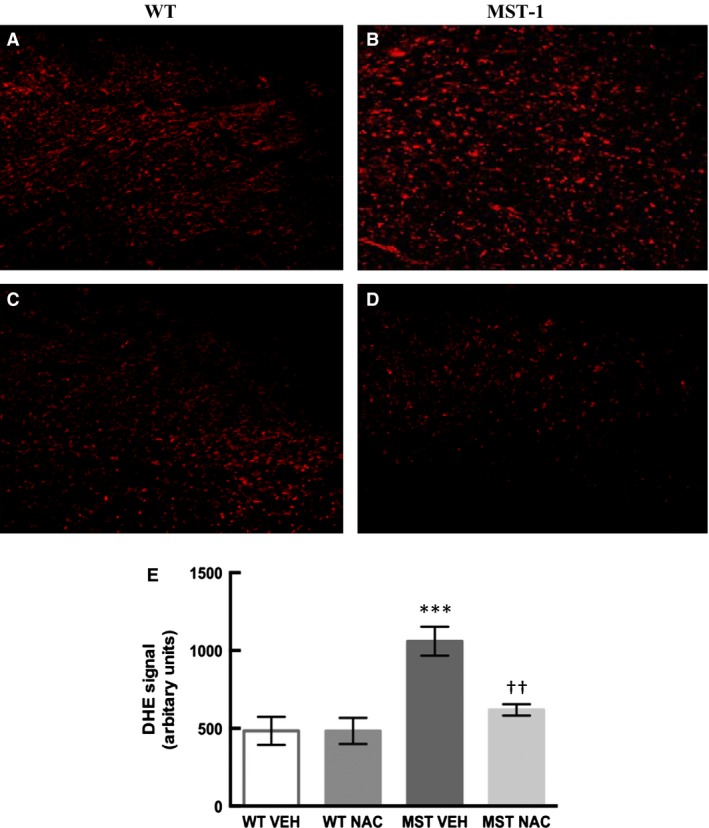
Representative images of dihydroethidium staining in frozen heart sections from (A) WT and (B) MST‐1 mice treated with saline vehicle and (C) WT and (D) MST‐1 mice treated with NAC. (E) Quantification of DHE signal in WT and MST‐1 mice treated with saline vehicle or NAC (*n *= 4–5). Cardiac oxidative stress as measured by DHE was greater in saline treated MST‐1 mice compared to saline treated WT. Treatment with NAC attenuated cardiac oxidative stress in MST‐1 mice. Data are mean ± SEM. ***P < 0.001 versus WT vehicle. ††*P* < 0.01 versus MST‐1 vehicle. DHE, Dihydroethidium. WT,wild‐type mice; MST‐1, transgenic mice with cardiac‐specific overexpression of MST‐1; NAC,* N‐*acetylcysteine; VEH, vehicle.

Cardiac oxidative stress as assessed by DHE was 119% greater in MST‐1 mice compared to WT mice (*P* < 0.001; Fig. 10). DHE staining was 42% less in MST‐1 mice receiving NAC compared to those receiving saline vehicle (*P* = 0.005; Fig. 10).

## Discussion

There were two major novel findings in the present study. Firstly, we found that cardiac perivascular and interstitial fibrosis and cardiac expression of collagen types I and III were greater in MST‐1 mice compared to WT mice. Of interest, we found that perivascular and cardiac interstitial fibrosis were less in MST‐1 mice treated with NAC compared to those treated with saline vehicle. Consistent with a reduction in cardiac fibrosis, cardiac collagen types I and III expression was also less in NAC‐treated MST‐1 mice than those treated with saline vehicle. These data provide direct evidence that NAC can prevent the development of cardiac fibrosis in the setting of HF. Together, these findings indicate that NAC can prevent the development of cardiac fibrosis in a mouse model of dilated cardiomyopathy. These data are in agreement with previous findings indicating cardioprotective effects of NAC in the settings of diabetes, hypertension, hypertrophic cardiomyopathy, and in acute cardiac injury (Rauchova et al. 2005; Marian et al. 2006; Sun 2007; Liu et al. 2015).

Secondly, we found that the thickness of the interventricular septum was greater in MST‐1 mice compared to WT mice. In NAC‐treated MST‐1 mice, the thickness of the interventricular septum was less compared to vehicle‐treated MST‐1 mice. These findings indicate that NAC treatment can attenuate HF‐dependent cardiac remodeling. Cardiac oxidative stress, as measured by DHE, was greater in MST‐1 mice compared to WT mice. In MST‐1 mice treated with NAC, cardiac oxidative stress was less compared to those treated with saline. Thus, the effects of NAC on cardiac fibrosis and related cardiac remodeling, at least in part, are likely to be mediated via its antioxidant effects. Ejection fraction and fractional shortening were less in MST‐1 mice compared to WT mice and NAC treatment had minimal effect on these parameters in MST‐1 mice suggesting that reduced cardiac fibrosis and remodeling observed following NAC treatment in this genotype was not sufficient enough to translate into improvements in cardiac function.

Cardiac fibrosis, particularly that in the interstitium, has been considered to play an important role in the pathogenesis of chronic HF (Pan et al. 2011; Edgley et al. 2012). Consequently, treatment interventions that can target cardiac interstitial fibrosis are of utmost significance. In this study, the level of cardiac interstitial fibrosis in MST‐1 mice was modest, but nonetheless, it was significantly greater than that observed in WT mice. In this context, it is important to note that even modest increases in cardiac interstitial fibrosis can contribute to cardiac dysfunction and indeed, we found that ejection fraction and fractional shortening were less in MST‐1 mice, compared to WT. Importantly, cardiac interstitial fibrosis was less in MST‐1 mice treated with NAC than those treated with saline. These findings indicate that NAC can attenuate the development of cardiac interstitial fibrosis in the setting of HF. Consistent with this, it has previously been shown that NAC has anti‐fibrotic effects in experimental hypertrophic cardiomyopathy (Marian et al. 2006; Lombardi et al. 2009).

We found that the amount of cardiac perivascular fibrosis in MST‐1 mice was much greater than that of interstitial fibrosis. While interstitial fibrosis is widely considered to play a direct role in the pathogenesis of HF as discussed above, the precise role of perivascular fibrosis in the development of HF remain to be determined. There is evidence that perivascular fibrosis is associated with reduced coronary perfusion in patients with nonischemic HF (Dai et al. 2012). As impaired coronary perfusion can in turn have a direct impact on cardiac function, these findings provide a basis for targeting perivascular fibrosis in the treatment of HF. We found that perivascular fibrosis was less in MST‐1 mice treated with NAC compared to those treated with saline. Thus, our findings provide evidence that NAC can reduce cardiac perivascular and interstitial fibrosis associated with HF. It is also of interest to note that NAC can reduce oxidative stress, improve cardiac index, and reduce mortality in patients with acute myocardial infarction (Simon and Szepvolgyi 1996). While these authors did not examine cardiac fibrosis, the beneficial cardiac effects of NAC observed in this study may have been mediated via its antifibrotic effects. Overall, these data provide evidence that NAC can provide cardioprotective effects to HF patients potentially via attenuation of cardiac fibrosis.

We found that the thickness of the interventricular septum during systole and diastole was less in MST‐1 mice compared to WT which was restored by NAC treatment. These data support our hypothesis that reducing oxidative stress can minimize cardiac remodeling in the setting of HF. Cardiac‐specific overexpression of MST‐1 induces apoptosis leading to cardiac remodeling (Yamamoto et al. 2003). The precise role of MST‐1 in human HF remains to be determined, but apoptosis plays a role in cardiac remodeling (Cesselli et al. 2001) and is relevant to the pathogenesis of human HF (van Empel et al. 2005). Importantly, there is evidence that oxidative stress can induce apoptosis which provides a basis to target oxidative stress to reduce apoptosis‐induced cardiac remodeling (Kumar et al. 2002). Our present findings suggest that NAC can attenuate cardiac remodeling associated with apoptosis potentially via reducing cardiac oxidative stress.

Oxidative stress plays a central role in the development of cardiac fibrosis regardless of the underlying etiology (Aragno et al. 2008; Zhao et al. 2008; Tsutsui et al. 2011). In addition to the pathogenic role of augmented oxidative stress in the development of cardiac fibrosis (Aragno et al. 2008; Zhao et al. 2008; Tsutsui et al. 2011), there is also evidence that oxidative stress plays a role in cardiac remodeling as alluded to above. Consequently, we determined whether the effects of NAC were associated with reduced myocardial oxidative stress. Cardiac oxidative stress as measured by DHE staining was greater in MST‐1 mice compared to WT. Of note, DHE staining was less in MST‐1 mice treated with NAC compared to those treated with saline. These data indicate that NAC can reduce cardiac oxidative stress which in turn may reduce cardiac fibrosis and remodeling in HF.

As blood pressure can have a direct impact on the development of cardiac fibrosis, we assessed blood pressure in WT and MST‐1 mice. As expected, blood pressure was less in MST‐1 mice with HF compared to WT mice, and NAC treatment had minimal effect on blood pressure. Thus, we can be confident that the anti‐fibrotic effects of NAC were mediated independently of any concomitant changes in blood pressure.

Ejection fraction, fractional shortening, dP/d*t*_max_, and d*P*/d*t*_min_ were less in MST‐1 mice compared to WT. NAC had minimal effect on these measurements despite its ability to attenuate cardiac fibrosis and remodeling in MST‐1 mice. This is not surprising as these mice have cardiac‐specific overexpression of MST‐1 (Yamamoto et al. 2003) and treatment interventions are unlikely to be able to mitigate the effects of this transgene. MST‐1 can induce apoptosis leading to dilated cardiomyopathy in mice. While apoptosis is present in human HF, overexpression of MST‐1 has not been documented and it remains to be determined whether NAC can mitigate cardiac fibrosis in the clinical setting (Yamamoto et al. 2003). Alternatively, echocardiography may not be sensitive enough to detect subtle improvements in cardiac function in MST‐1 mice.

Cardiac expression of BNP and *β*‐MHC was greater in MST‐1 mice compared to WT, and NAC treatment had minimal effect on these measurements suggesting that the presence of cardiac injury was not completely alleviated in MST‐1 mice treated with NAC. It may be that NAC fails to mitigate cardiac injury arising from cardiac overexpression of MST‐1 and subsequent apoptosis in these mice.

In conclusion, our findings provide direct evidence that NAC can attenuate cardiac fibrosis and remodeling in the setting of HF. As these factors play major pathological roles in the development of HF, our current findings provide a solid basis to further investigate the effects of NAC in HF.

## Conflict of Interest

None declared.
